# Editorial: Pharmacology and treatment methods for NAFLD in the context of diabetes

**DOI:** 10.3389/fendo.2022.1010485

**Published:** 2022-09-01

**Authors:** Mohamed Abu-Farha, Yong-ho Lee, Jehad Abubaker

**Affiliations:** ^1^ Biochemistry and Molecular Biology Department, Dasman Diabetes Institute, Dasman, Kuwait; ^2^ Department of Internal Medicine, Yonsei University College of Medicine, Seoul, South Korea; ^3^ Institute of Endocrine Research, Yonsei University College of Medicine, Seoul, South Korea; ^4^ Department of Systems Biology, Glycosylation Network Research Center, Yonsei University, Seoul, South Korea

**Keywords:** NAFLD, GLP1 agonist, SGLT2 (sodium-glucose cotransporter 2) inhibitor, thiazolidinediones, liver fat

Non-alcoholic fatty liver disease (NAFLD) is a condition characterized by significant fat accumulation in the liver in the absence of excessive alcohol consumption. This can result in hepatic steatosis and progress to fibrosis and liver cirrhosis. Genetics factors, lifestyle, hormonal factors, and insulin resistance are all known to contribute to NAFLD development. As such, the rise in prevalence follows the trend of obesity and diabetes. NAFLD is highly prevalent in patients with type 2 diabetes (T2D) affecting up to 60% of diabetic patients. The presence of NAFLD has also been associated with diabetic complications such as kidney damage and retinopathy. Similarly, T2D has been shown to result in poor outcomes for NAFLD patients. As both diabetes and NAFLD may share a causal relationship, each is a risk factor for the other.

Therefore, in tackling the rising pandemic of diabetes, the treatment of NAFLD and diabetes is an essential public health matter. Due to their close relationship, effective treatment methods targeting one condition can improve the outcomes for the other. For example, the use of hyperglycemic agents in treating insulin resistance and hyperinsulinemia has been used as a way for preventing the progression of NAFLD. This special issue includes four meta-analyses that looked at various hypoglycemic agents in the treatment of NAFLD and T2D with special focus on thiazolidinedione (TZD), sodium-glucose co-transporter-2 (SGLT2) inhibitors as well as glucagon-like peptide-1 receptor agonists (GLP1-RA).

In the first analysis, Lian and Fu performed a network meta-analysis to evaluate the efficacy of various hypoglycemic agents in the treatment of NAFLD in the absence or presence of diabetes. After looking at multiple studies, they concluded that thiazolidinediones, especially pioglitazone, is beneficial in normalizing glucose metabolism in NAFLD patients, but its use was associated with weight gain. GLP1-RAs and metformin were also associated with improved ALT and AST levels in people with NAFLD. They further highlighted the possibility of combining pioglitazone with other weight loss drugs such as GLP1-RA that can mitigate the weight gain and further provide advantages in terms of reducing the metabolic burden in patients diagnosed with NAFLD and diabetes. In the second analysis also by Lian and Fu, they further looked at the usage of pioglitazone for NAFLD patients with prediabetes or T2D. Their analysis further emphasized the positive effect of pioglitazone on NAFLD in the presence or absence of diabetes. They showed based on their literature analysis that pioglitazone had a significant effect on improving liver histology, such as steatosis, inflammation, and ballooning, but not fibrosis. Additionally, they highlighted the beneficial effect of pioglitazone in terms of improving insulin sensitivity, hyperglycemia, as well as AST and ALT levels, recommending its usage as a first line NAFLD therapy. The analysis also discussed the side effects commonly associated with the usage of pioglitazone, including weight gain, chronic lower limb edema, and back or joint pain.

Zhu et al. further investigated the efficacy and safety of GLP1-RA in patients with T2D and NAFLD, given the increased usage of the drug in this population (i.e. NAFLD patients who are also diabetic). In all, eight trials were eligible for inclusion in the review, which made up a total of468 participants with both T2D and NAFLD. Overall, people taking GLP1-RA showed improvement in all outcome measures which included decreased intrahepatic, subcutaneous, and visceral adipose tissue. A significant improvement was also observed in secondary outcomes including reduction in ALT, AST, and BMI, amongst other clinical parameters. Given the mild adverse events associated with GLP1- RA, the authors concluded that GLP1-RA was a highly effective treatment for NAFLD, especially in people with T2D.

Finally, another diabetic medication, SGLT2i, was assessed by Wong et al. They specifically investigated the effectiveness of SGLT2i usage for treatment of NAFLD in patients with T2D in Asian patients. They showed that SGLT2i was effective in improving hepatic steatosis and to some extent even liver fibrosis. In conclusion, all the above studies showed the potential positive impact of utilizing anti-diabetic medications in the treatment of NAFLD.

## Author contributions

All authors listed have made a substantial, direct, and intellectual contribution to the work and approved it for publication.

## Conflict of interest

The authors declare that the research was conducted in the absence of any commercial or financial relationships that could be construed as a potential conflict of interest.

## Publisher’s note

All claims expressed in this article are solely those of the authors and do not necessarily represent those of their affiliated organizations, or those of the publisher, the editors and the reviewers. Any product that may be evaluated in this article, or claim that may be made by its manufacturer, is not guaranteed or endorsed by the publisher.

